# Surgical Treatment for Isolated Tricuspid Valve Disease: A Less Invasive Approach for Better Outcomes

**DOI:** 10.3390/jcm13113144

**Published:** 2024-05-27

**Authors:** Cristina Barbero, Marco Pocar, Dario Brenna, Andrea Costamagna, Valentina Aloi, Cecilia Capozza, Claudia Filippini, Anna Chiara Trompeo, Stefano Salizzoni, Luca Brazzi, Mauro Rinaldi

**Affiliations:** 1 Department of Cardiovascular Surgery, Città della Salute e della Scienza, University of Turin, 10126 Torino, Italy; marco.pocar@unito.it (M.P.); dbrenna89@gmail.com (D.B.); valentina.aloi@unito.it (V.A.); cecicap@hotmail.it (C.C.); stefano.salizzoni@unito.it (S.S.); mauro.rinaldi@unito.it (M.R.); 2 Division of Cardiac Intensive Care, Anesthesia, Intensive Care and Emergency Department, Città della Salute e della Scienza, University of Turin, 10126 Torino, Italy; andrea.costamagna@hotmail.it (A.C.); claudia.filippini@unito.it (C.F.); annachiara.trompeo@unito.it (A.C.T.); luca.brazzi@unito.it (L.B.)

**Keywords:** tricuspid valve, right ventricle, pulmonary hypertension, TRI-SCORE, late referral, operative mortality

## Abstract

**Background**. Severe tricuspid valve (TV) disease has a strong association with right ventricle dysfunction, heart failure and mortality. Nevertheless, surgical indications for isolated TV disease are still uncommon. The purpose of this study is to analyze outcomes of patients undergoing minimally invasive isolated TV surgery (ITVS). **Methods**. Data of patients undergoing right mini-thoracotomy ITVS were prospectively collected. A subgroup analysis was performed on late referral patients. Five-year survival was assessed using the Kaplan–Meier survival estimate. **Results**. Eighty-one consecutive patients were enrolled; late referral was recorded in 8 out of 81 (9.9%). No cases of major vascular complications nor of stroke were reported. A 30-day mortality was reported in one patient (1.2%). Five-year Kaplan–Meier survival analysis revealed a significant difference between late referral patients and the control group (*p* = 0.01); late referral and Euroscore II were found to be significantly associated with reduced mid-term survival (*p* = 0.005 and *p* = 0.01, respectively). **Conclusions**. To date, perioperative mortality in patients undergoing ITVS is still consistently high, even in high-volume, high-experienced centres, and this accounts for the low rate of referral. Results from our report show that, with proper multidisciplinary management, appropriate pre-operative screening, and allocation to the safest approach, ITVS may offer better results than expected.

## 1. Introduction

The prevalence of primary and secondary significant tricuspid valve (TV) disease is increasing in the general population, and to date, it is estimated to be up to 3% after the age of 75, with a well-known association with mortality and morbidity [1,2].

Despite poor prognosis for untreated TV disease, surgical management is routinely planned only at the time of left-sided valve surgery (LSVS), and patients are addressed for isolated tricuspid valve surgery (ITVS) mainly in case of advanced disease with significant right ventricle dysfunction [3]. The low trend of patient referral for ITVS is essentially triggered by suboptimal post-operative outcomes with a mortality rate still ranging from 3% to 16% even in contemporary practice and high-volume centers [4,5,6,7]. At the same time, unexpected poor results have been reported also by transcatheter TV repair experiences [8,9,10]. As a consequence, interest in better understanding this challenging disease, identifying risk factors for poor prognosis after treatment, and identifying the safest and most effective approach is increasing.

The burden of associated heavy comorbidities—such as history of coronary disease, previous LSVS, and chronic kidney and liver disease—of patients undergoing ITVS and the advanced right ventricle dysfunction at referral are definitely the main reasons that account for these lacking results [5]. However, recent evidences in the literature are focusing on the role that different surgical strategies have on early outcomes. When looking at the type of surgical approach, the right mini-thoracotomy reducing the need for extensive opening of the pericardium may prevent further deterioration of right ventricular function, may reduce the risk of heart and great vessels injuries during re-sternotomy in case of redo procedures, may reduce the risk of functional decline after prolonged intensive care unit (ICU) and hospital length of stay, and may offer the patient an easier post-operative course with early return to home.

The present study analyses our more than 15-year experience in minimally invasive ITVS focusing on early and late outcomes.

## 2. Materials and Methods

### Study Design and Population

Between 2006 and 2022, 300 patients with TV disease underwent minimally invasive surgery at our department. Among these, 81 patients (27%) received ITVS. Data collection was prospectively performed and approved for research by the Institutional Review Board of the University of Turin (protocol number 0047596, 29 April 2021). Consent of patients has been waived.

Inclusion criteria were diagnosis of isolated TV disease and surgical indication for a right mini-thoracotomy approach. Exclusion criteria were concomitant LSVS, coronary artery bypass surgery, ascending aorta surgery, atrial septal defect closure, and contraindication to the minimally invasive approach.

In our everyday clinical practice, patients with isolated TV disease are accurately discussed in a multidisciplinary heart team made of cardiac surgeons, cardiac anesthesiologists, and cardiologists to ponder the indications for surgery, evaluate the risk profile of the patient, and consider potential alternatives to surgery such as optimization of the medical therapy and/or micro-invasive procedures. Then, patients considered suitable for surgery are screened for the minimally invasive approach, which is the standard of care for TV surgery at our department. The pre-operative screening consists of a computerized tomography scan and/or aorto-iliac-femoral vessels angiography for the diagnosis and grading of atheromatous disease; a right heart catheterization to assess the degree of right ventricle dysfunction and of pulmonary hypertension, and a coronary angiography. Patients enrolled for surgery are managed with a minimally invasive protocol consisting of a right mini-thoracotomy approach, a full peripheral cannulation, and tailored perfusion strategies and aortic clamping techniques; when possible, TV surgery is performed on a beating heart. The minimally invasive approach allows shorter ventilation times, shorter ICU length of stay, early removal of drains, early mobilization, and faster return to home, which is particularly important in such fragile patients [11].

Then, the patient is allocated to the most appropriate arterial perfusion setting and aortic clamping technique [12,13]. Operative risk was stratified according to EUROscore and TRI-SCORE models [14,15]. Aggressive preoperative hemodynamic optimization, with a particular focus on right ventricular function and pulmonary hypertension, was performed throughout the study period.

Long-term outcomes were obtained from post-operative follow-up visits at the outpatient clinic, or from telephone interviews.

## 3. Definitions

Referral to surgery was considered late if the patient had a non-elective surgery status (transferred from the emergency department or from another acute care facility), or was admitted with acute heart failure or advanced liver disease, or had an unplanned hospitalization within the 90 days before surgery [16]. Stroke was defined as neurological signs persisting at the time of discharge from the hospital and/or in the presence of localized ischemic infarcts detectable by conventional neuroimaging techniques. Operative mortality was defined as death occurring during hospitalization or within 30 days from the index procedure.

### 3.1. Surgical Technique [12,13,17]

All patients underwent surgery through a right mini-thoracotomy in the fourth intercostal space and double lumen endotracheal tube to allow single-lung ventilation. A soft tissue retractor was used to expose the main surgical port, and an endoscope was inserted in an accessory port created below the working port; the same port is used for carbon dioxide insufflation. An additional sixth intercostal space port is created for pump suction. Intraoperative trans-esophageal echocardiography was used in all the patients for a pre-operative anatomical analysis of the valve, and to guide cannulas positioning. After full heparinization, peripheral cardiopulmonary bypass was established. Arterial perfusion strategies and aortic clamping techniques used during the study period were retrograde arterial perfusion (RAP) with an endo-aortic clamp (EAC), RAP with a trans-thoracic clamp (TTC), antegrade arterial perfusion (AAP) through the axillary artery with TTC, and RAP or AAP with a beating heart. The choice of one setting in respect to the others was, in part, orientated on TV anatomy and complexity of the surgical procedure, and was in part dependent on the clinical history and vascular anatomy of the patient (i.e., previous cardiac surgery, peripheral vasculopathy). Venous return was routinely obtained with a double cannulation (jugular and femoral). Caval snaring was obtained by placing tourniquets around the superior and inferior venae cavae or by placing endovascular balloons (7F, 65 cm; Boston Scientific, Natick, MA, USA). Endovascular balloons were placed under transesophageal echocardiography into the right atrium from the right femoral vein (through a 7F introducer) and from the right jugular vein (through a Y-modified cannula); this last approach was the preferred in redo cases to avoid demanding dissections. When required, Histidine-tryptophan-ketoglutarate (Custodiol^®^, Alsbach-Hähnlein, Germany) or St. Thomas 1 crystalloid solutions were used for myocardial protection.

### 3.2. Statistical Analysis

Descriptive data were tested for normal distribution by the Shapiro–Wilk test and presented as mean and standard deviation (SD) or median and interquartile range (IQR) (continuous variables), and as numbers and percentages (categorical variables) as appropriate. A further analysis was performed comparing the subgroup of late referral patients with the control group of the other patients. Five-years survival was assessed using the Kaplan–Meier survival estimate, and the log-rank test was used to analyze differences in the survival curves among groups (late referral and control group). The univariable Cox regression model of survival was used to test baseline and intraoperative variables associated with 5-year survival, and the univariable predictors with a *p* < 0.05 were included in the multivariate regression model. To assess proportional hazards assumption violations, a proportional-hazards assumption test was performed on the Cox multivariable model based on Schoenfeld residuals. Statistical analyses were performed using Stata 16.1/SE (Stata Corporation, College Station, TX, USA).

## 4. Results

During the study period, 81 consecutive patients underwent right mini-thoracotomy ITVS. Baseline characteristics and details on diagnosis are reported in Table 1.

The mean age was 62 ± 16.1 years, and more than 60% were female. Fifty-seven patients had undergone previous cardiac surgeries (70.4%); of these, more than 50% had a history of LSVS. Previous surgery on the TV was reported in 15 out of 57 patients (26.3%). Most frequent causes of disease were organic (58% for 47 patients) and functional (35.8% for 29 cases); tricuspid prosthesis dysfunction was reported in 4 cases (4.9%), while valve regurgitation relapse in previous TV repair in 11 cases (13.5%). Late referral was recorded in 8 out of 81 patients (9.9%), including 4 patients admitted with acute decompensated heart failure, and 4 patients with advanced liver disease.

Operative data and post-operative outcomes are given in Table 2.

Fifty-one patients underwent TV replacement (62.9%), while a conservative procedure was feasible in 30 patients (37.1%). Of the 51 patients undergoing TV replacement, 50 received biological prostheses (98%).

In most of the patients, CPB was established with RAP through the femoral artery (98.8%, 80 patients), and in most of the cases, a beating heart procedure was performed (62.9%, 51 patients). Conversion to sternotomy was required in 2 cases (2.5%) due to uncontrolled bleeding from the left auricle in the first case, and to uncontrolled bleeding from the right ventricle in the second case.

No cases of major vascular complications nor of stroke were reported. Re-exploration for bleeding was necessary in seven patients (8.6%); all these patients underwent re-exploration through the right mini-thoracotomy. Median ICU and in-hospital length-of-stay were 1 and 7 days, respectively. A 30-day mortality was reported in one patient with advanced right ventricular dysfunction and liver disease at admission (1.2%).

**Figure 1:**
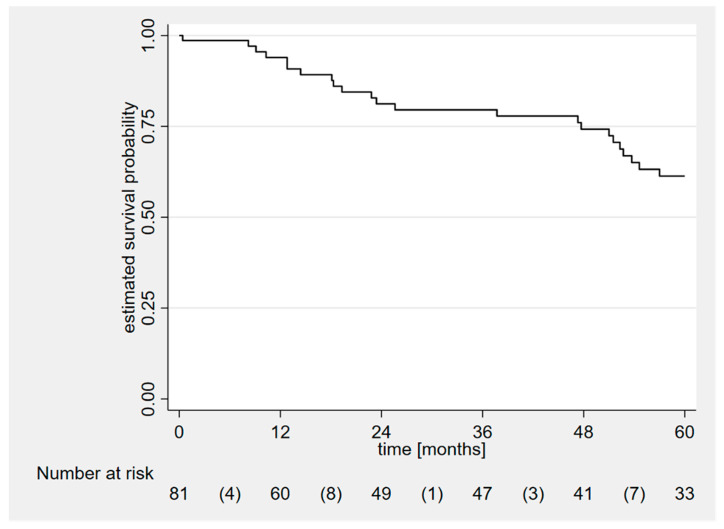
Kaplan–Meier survival curve for a single group (total follow up time: 5 years). The event of interest was death. Time is plotted on the X-axis as months and probability of survival on the Y-axis.

**Figure 2:**
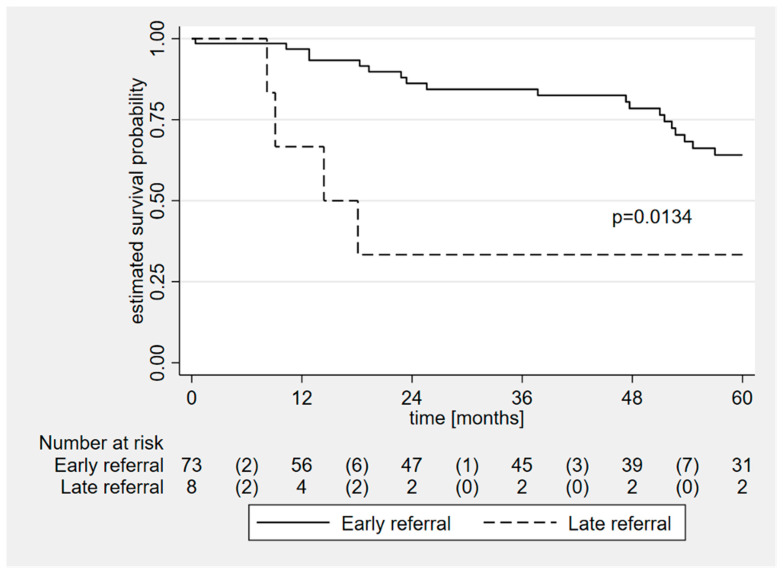
Kaplan–Meier survival curves for the early and late groups (total follow up time: 5 years). The event of interest was death. Time is plotted on the X-axis as months and probability of survival on the Y-axis.

Median follow-up was 48 months (range: 3–204). The overall Kaplan–Meier 5-year survival estimate is shown in Figure 1: a total of 4 (5%), 12 (15%), and 23 (28%) patients died at 1, 2, and 5 years from surgery, respectively. In the subgroup of late referral patients, 1, 2 and 5-year mortality was higher than in the control group: 2 (25%), 4 (50%) and 4 (50%) vs. 2 (3%; *p* = 0.047), 8 (11%; *p* = 0.015) and 19 (26%; *p* = 0.214) patients, respectively (Table 2); the 5-year Kaplan–Meier survival analysis was significantly different between groups (Figure 2; *p* = 0.0134).

All the preoperative and intraoperative variables were tested in a univariable Cox regression model: late referral, chronic kidney disease, coronary artery disease, and EuroSCORE II were found to be significant. At the multivariable Cox regression model, built on these four variables, late referral and EuroSCORE II remained significantly associated with reduced long-term survival (*p* = 0.005 and *p* = 0.010, respectively; Table 3).

Age, TRI-SCORE and preoperative eGFR tested significative at the univariate analysis but were excluded to the multivariate model because of collinearity with the EuroSCORE II variable. All the variables tested respected the proportional hazards assumptions in the proportional-hazards assumption test performed on the Cox multivariate model based on Schoenfeld residuals.

## 5. Discussion

Chronic severe TV disease is associated with a two-fold increase in cardiac mortality in the general population, mainly due to the progressive development of right ventricular remodeling and dysfunction [18,19,20,21]. At the same time, evidence in the literature shows that patients are rarely and late referred for surgery, and that outcomes after surgery are still unsatisfactory even in updated reports from high volume, high-experienced centers [5,22,23,24]. In a recent report by Dreyfus J. and colleagues, 466 ITVS from 12 French centers were analyzed: the in-hospital mortality was 10%; NYHA class III or IV, heart failure symptoms, low prothrombin time, and moderate to severe right ventricular dysfunction were found to be independent risk factors for early mortality. The authors emphasized the importance of the timely referral of patients with severe TV disease for intervention [24]. Kawsara et al. analyzed more than 1500 ITVS patients from 28 centers in the United States and reported an in-hospital mortality rate higher than 8%; surrogates of late referral were frequent, 41% of patients were admitted with acute heart failure, 44% had a non-elective surgery status, 17% had advanced liver disease, and 31% had at least one unplanned hospitalization in the 90 days before the index surgery hospitalization. Again, the authors have clearly demonstrated that late referral is a strong independent predictor of in-hospital mortality in patients undergoing ITVS (OR 4.75; 95% CI 2.74–8.25), and they suggest this should be definitely taken into consideration when planning a surgical procedure in such fragile patients [16]. Then, a current report by Axtell et al. showed that ITVS in patients with decompensated heart failure did not improve survival when compared to a propensity-matched cohort of patients treated medically [25]. Reasons for this highly reported late referral in patients with a clear indication for TV surgery are likely multifactorial. First, patients with isolated TV disease may be asymptomatic for a long time, and when symptoms occur, they are usually not specific. Second, right ventricle function and size are challenging to evaluate during routine visits and echocardiographic examinations. Third, current guidelines for the management of heart valve diseases do not really support indications for surgery in patients with isolated TV disease. The American Heart Association/American College of Cardiology guidelines assigns a class IIA recommendation for ITVS, and only in patients with signs and symptoms of decompensated heart failure and with signs of right ventricular dilation and dysfunction [3]. Finally, even in the contemporary era, surgery for isolated TV disease remains associated with considerable peri-operative morbidity and mortality, and this may deter physicians and cardiologists from referring [5,22,24,26,27].

In this contest, the impact of surgical techniques on the prognosis of these high-risk patients should not be ignored. The right mini-thoracotomy approach, avoiding wide opening of the pericardium, may prevent further deterioration of right ventricular function, may reduce the risk of heart and great vessels injuries during re-sternotomy in case of previous cardiac surgery procedures, may reduce the risk of functional decline due to a prolonged ICU and hospital length of stay in fragile patients, and may offer the patient an easier post-operative course with early return to home [15,28].

A cohort of 81 ITVS high-risk patients—mean logistic EuroSCORE 10.3%, mean EuroSCORE II 5.9, TRI-SCORE 3.6, 44% of chronic kidney disease, 16% of chronic liver disease, more than 70% of previous cardiac surgery, and nearly 50% of these with previous surgery on the mitral valve—is analyzed here. The main result of our analysis is a low 30-day mortality when compared to reports existing in the literature on patients undergoing ITVS through a standard sternotomy (1.2% vs. 8–10%) [5,22,29,30]. Moreover, good results are also stated in terms of major vascular and neurological complications (no cases reported in the present series), and ICU and post-operative length of stay (median 1 and 7 days, respectively). In the subgroup analysis, 1 and 2-year mortality were significantly higher in the late referral patients; particularly, the two curves diverged after 12 months, and at that time-point, 3 late referral patients and 1 patient in the control group reported relapse with moderate to severe TV regurgitation.

Our results are in line with a report by Chen et al. on 107 patients undergoing minimally invasive ITVS after previous LSVS; the reported operative mortality rate was 3.7%, and authors concluded that the notoriously high mortality of ITVS can partially be attributed to the surgical approach [31]. As the low peri-operative mortality rate reported, we were unable to recognize predictors of peri-operative mortality. However, when looking at mid-term follow-up, Kaplan–Meier survival analysis clearly showed a significant difference between late referral patients and the control group (*p* = 0.01); then, late referral and a high EuroSCORE II were found to be significantly associated with reduced long-term survival (*p* = 0.005 and *p* = 0.01, respectively).

The good results of this report can be explained considering our more than 15-year experience on different minimally invasive settings of arterial perfusion and aortic clamping that are routinely tailored on the single patients according to baseline characteristics and vascular anatomy. The meticulous pre-operative vascular screening allows surgeons to direct patients towards the safest approach and reduce the risk of neurological or vascular complications triggered by retrograde arterial perfusion and retrograde catheter manipulation in atheromatous arteries. Furthermore, a landmark of our heart-team management is to direct patients with significant TV disease towards surgery before the onset of symptoms or right ventricle dysfunction. In the present cohort, the rate of late referral is 9.9% for 8 patients, and again, this is definitely lower than the rates reported in the literature [24,25]. The only case of 30-day mortality reported in this series occurred in a late referral patient admitted to the emergency department with acute decompensated heart failure.

In our follow-up data, late referral also remains a significant risk factor for mortality after a relatively long period of time after the surgical procedure.

These findings support the hypothesis that ITVS should not be considered a high-risk procedure per se, as it is largely perceived, since poor outcomes are likely explained by the high-risk profile of patients who are referred to surgery at a very late stage of the disease. Moreover, a minimally invasive approach should be considered a useful tool able to improve outcomes in this fragile subgroup of patients.

## 6. Limitations

The main limitation is the single center, retrospective design of the study. Furthermore, only a low amount of data on right ventricle function and dimension was reported, so details regarding adherence to the ESC guidelines were not available. The lack of a control group of patients undergoing ITVS through a standard sternotomy is another limitation; however, the standard of care at our institution for TV surgery is the right mini-thoracotomy approach, so a proper control group was not accessible.

## 7. Conclusions

To date, perioperative mortality in patients undergoing ITVS is still consistently high, even in high-volume, highly experienced heart valve centres, and this is consistent with the low rate of referral to surgery.

In contrast with the bulk of data reported in the literature, results from our more than 15-year experience in minimally invasive valve surgery show that, with proper multidisciplinary management in the heart-team, an appropriate pre-operative vascular screening, and patient allocation to the safest approach, ITVS is able to offer better results than expected.

## Figures and Tables

**Table 1:** Baseline patients’ characteristics and echocardiographic data.

	*n* = 81
Female gender	51 (62.9)
Age (y)	62 (16.1)
BMI (kg/m^2^)	24 (3.6)
Hypertension	52 (64.2)
Diabetes	18 (22.2)
Creatinine (mg/dL)	1.4 (1)
Estimated CrCl (mL/min)	57.3 (30.7)
CKD	36 (44.4)
COPD	3 (3.7)
Chronic liver disease	13 (16)
Peripheral vasculopathy	5 (6.2)
Neurological deficit	4 (4.9)
Atrial fibrillation	52 (64.2)
NYHA ≥ 3	48 (59.3)
Log EuroSCORE (%)	10.3 (10.5)
EuroSCORE II	5.9 (8.6)
TRI-SCORE	3.6 (2.6)
Late referral	8 (9.9)
Previous cardiac surgery	57 (70.4)
Previous MV surgery	27 (47.4)
Previous TV surgery	15 (26.3)
Previous heart transplant	9 (11.1)
More than 1 previous cardiac surgeries	19 (40.4)
Organic TV disease	47 (58)
Infective endocarditis	7 (8.6)
Functional TV disease	29 (35.8)
Prosthesis degeneration	4 (4.9)
Left ventricle ejection fraction (%)	57.5 (8.8)
Moderate/severe TV regurgitation	75 (92.6)
Moderate/severe TV stenosis	6 (7.4)
TV annulus diameter (mm)	45.7 (6.9)
PAPs (mmHg)	42.5 (11.4)

**BMI**: body mass index; **CrCl**: creatinine clearance; **CKD**: chronic kidney disease; **COPD**: chronic obstructive pulmonary disease; **NYHA**: New York Heart Association; **Log**: logistic; **MV**: mitral valve; **TV**: tricuspid valve; **PAPs**: systolic pulmonary artery pression. Data are presented as median (SD) or number (rate) as appropriate.

**Table 2:** Intraoperative data and post-operative outcomes.

	*n* = 81
**Retrograde arterial perfusion**	80 (98.8)
**Beating heart TV surgery**	51 (62.9)
**Endo-aortic clamp**	18 (48.6)
**Trans-thoracic clamp**	12 (51.4)
**TV replacement**	51 (62.9)
**TV repair**	30 (37.1)
**AF ablation**	2 (2.5)
**CPB time** (min)	103.8 (29.6)
**Clamping time** (min)	62.8 (24.1)
**Conversion to full sternotomy**	2 (2.5)
**Intra-operative aortic dissection**	-
**Re-exploration for bleeding**	7 (8.6)
**Stroke**	-
**Acute myocardial infarction**	-
**Acute kidney injury**	2 (2.5)
**Mechanical ventilation** (h)	17.2 (22.4)
**Mechanical ventilation >72 h**	3 (3.7)
**Permanent PM implantation**	5 (6.2)
**ICU stay** (days)	1 (1–3)
**New onset atrial fibrillation**	6 (7.4)
**Hospital stay** (days)	7 (6–12)
**30-day mortality**	1 (1.2)
**Moderate to severe TV regurgitation**	7 (8.6)
**PAPs** (mmHg)	36.3 (9.6)
**TV reoperation**	1 (1.2)

**TV**: tricuspid valve; **AF**: atrial fibrillation; **CPB**: cardiopulmonary bypass; **PM**: pace-maker; **ICU**: intensive care unit; **PAPs**: systolic pulmonary artery pression. Data are presented as median (SD), number (rate) as appropriate, or median (IQR).

**Table 3:** Uni- and multi-variate adjusted Cox regressions for 5-year survival.

Independent Variables	Multivariate Cox Regression				
	HR	SE	Z	CI 95%	*p*
Late referral	5.70	3.41	2.90	1.76–18.44	0.004
History of CAD	2.26	1.22	1.52	0.79–6.51	0.130
Chronic kidney disease	1.92	0.95	1.31	0.72–5.08	0.192
Euroscore II	1.09	0.04	2.32	1.01–1.18	0.021

**CAD**: coronary artery disease (treated); **HR**: hazard ratio; **SE**: standard error; **CI**: confidence interval.

## Data Availability

The data presented in this study are available on request from the corresponding author.
